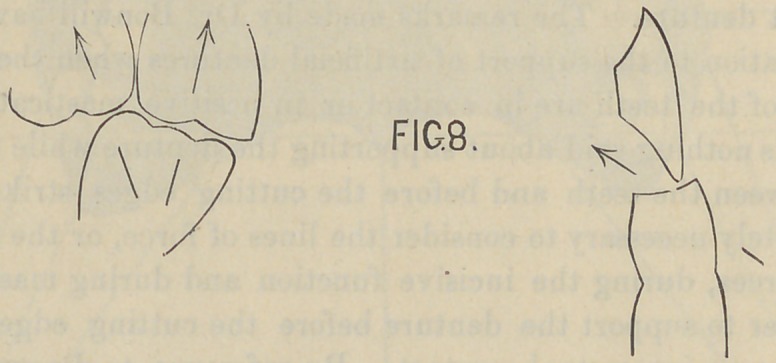# Some Points on the Articulation of Teeth

**Published:** 1894-03

**Authors:** G. Molyneaux

**Affiliations:** Cincinnati, O.


### Some Points on the Articulation of Teeth.

BY G. MOLYNEAUX, D.D.S., CINCINNATI, O.

Read before the Ohio State Dental Society, December, 1893.

The downfall of mechanical dentistry has been attributed to two causes, namely: the introduction of vulcanite for artificial dentures and nitrous oxide gas. The deplorable condition into which mechanical dentistry has fallen through vulcanite is not due so much to the fact that vulcanite as a base for artificial dentures is bad, for it is not, but to the fact that from the ease of manipulating this material a large number of persons have entered this profession who are entirely oblivious to anything more than jewelry work, losing sight entirely of the essential features—those features which make a denture of the greatest utility and beauty.

Of the many offices of the natural teeth there is none of greater importance than the mastication of the food. The thorough exercise of the teeth in mastication has been deemed a necessary concomitant to the process of digestion, and it is necessary, in order to maintain the health and comfort of the digestive organs, that this function shall be thoroughly performed. If in the insertion of an artificial denture we fail to provide a means for the thorough mastication of the food, so far as practical in each case, our instrument is virtually a failure. The means for properly masticating the food infers a perfect articulation of the teeth; not only the articulation of the teeth during the occlusion of the jaws, but also during the various movements of the lower jaw in the acts of incising and grinding the food. The lower jaw, on account of its peculiar form and movements, has the power of bringing the teeth into various relations to each other for the purpose; first, of incising the food, when both condyles of the lower jaw move forward in the glenoid fossæ, and the cutting edges of the incisor teeth are brought opposite each other for the purpose of separating a small portion of food from the main bulk prior to the process of mastication.

During mastication, instead of moving both condyles, one condyle only moves forward in the glenoid fossa protruding the lower jaw either to one side or the other, and this lateral protrusion followed by a drawing of the jaw back into the position of occlusion is a provision whereby the cusps of the bicuspid and molar teeth, if they are in proper relation to each other, can be utilized for the trituration of the food. It is an impossibility for the human race to masticate upon both sides of the mouth at the same time, and yet I have heard many practitioners in explaining to patients how to use an artificial denture, when they complain of tilting of the plates, that they must learn to chew on both sides at the same time.

It is a fact owing to careless methods in vogue that the movements of the lower jaw, for the purpose of mastication, with a large per cent. of artificial dentures is restricted simply to the up and down movement, or the same movement as in occlusion, and when the patients learn to control the lower jaw and make only the movements of occlusion they only then begin to feel that the denture is a success. By such movement of the jaw instead of mastication the patient is limited to only a mashing of the food and the swallowing without the proper insalivation, which occurs during the free and unrestricted use of the lower jaw. Now, to construct an artificial denture that will permit of the free and unrestricted use of the lower jaw several points must be carefully considered, and these cannot be better presented than in the language of the author of a system of articulating artificial teeth, which I am expected to demonstrate this evening. Dr. Bonwill, in an article entitled the Geometrical and Mechanical Laws of the Articulation of the Human Teeth, found in the "American System of Dentistry," pages 496 to 498 inclusive, calls attention to a few conditions which he says exist in every normally articulated natural denture, and with a special apparatus of his own invention called the Anatomical Articulator he applies his conclusions to the arrangement of artificial teeth. His first important observation is with reference to the shape of the lower jaw, it being of a peculiar tripod arrangement and forming an equilateral triangle, "From the center of one condyle to the center of the other four inches is about the average, and it will also be found that from the center of each condyloid process to the median line at a point where the inferior centrals touch at the cutting edge is also four inches." These angles, he claims, vary never more than half an inch, which would make little difference in describing the arc of a circle. "No matter what the width from one condyloid process to the opposite process the distance is the same from the processes to the median

Fig. 9.

line of the lower jaw at the cutting edge of the central incisor teeth. The jaw forms a perfect triangle, for the purpose of bringing into contact the largest amount of grinding surfaces of the bicuspids and molars, and at the same time have the incisors on one side at once come into action during these lateral movements. It will also be found that from the cuspids, the bicuspids and molars run in nearly a straight line (Fig. 9) instead of a circular one, back towards the condyloid process, enabling them to keep the largest amount of surface always presented for mastication. The next important observation has reference to the position and relation of the incisor teeth. The upper incisors should overjut the lower incisors, while the lower have a corresponding underbite; without this arrangement the incisor teeth would lose their function. Were the incisors to strike

Fig. 11

directly upon each other the power to cut off food would be very much lessened. The normal arrangement of the incisor teeth is shown in Fig. 11. Now, where there is an overbite and an underbite of the incisor teeth, just in proportion to their depth will be the length of the cusps of the cuspids, bicuspids and molars."

The next observation has reference to the curvature of the teeth in the jaw, which is formed by a dipping down of the second bicuspid and shortening of the posterior cusp, with a turning upward of the second and first molar teeth toward the condyle of the jaw, as illustrated in Fig. 17, which is reproduced from a life-size engraving from Black's Dental Anatomy. This vertical curvature " commences at the first molar tooth, although it shows itself slightly at the bicuspid, practically it need only commence

Fig. 17.

at the first molar, and this curvature is proportioned to the underbite and overbite of the incisor teeth." The purpose of this curvature at the ramus, as shown in Figs. 14 and 15, is obvious ; when the lower jaw is protruded during the incisive function the molar teeth are in contact at the same time as the cutting edge of the incisor teeth, while the cuspids and bicuspids swing free. This arrangement prevents anything more than mere contact of the incisor teeth, and applying the principle to the arrangement of artificial teeth it establishes a contact at the heel of the plate at the same time that the incisor teeth strike, and prevents the displacement of the dentures. The length of the cusps of the bicuspid and molar teeth has a definite relation to the lateral movement of the lower jaw. The buccal cusps of the lower bicuspids and molar teeth are usually shorter than the lingual cusps, while the reverse is true of the upper bicuspids and molars. The purpose of this arrangement can be easily seen by referring to Fig. 12. When the lower jaw is protruded to the left'side as in the figure,

Fig. 14.

and mastication is being performed on the left side of the mouth, the cusps, both the lingual and buccal, are found opposite each other while the food is pressed into the sulcus between these cusps. Now, if there is no contact upon the opposite side of the mouth the plate would be in great danger of tilting or dropping

Fig. 15.

on the side opposite the one in use. But, by the proper shaping of the cusps of the bicuspids and molars we find that we can obtain upon the opposite side of the mouth contact between the lingual cusps of the upper molar teeth and buccal cusps of the lower molars, which prevents displacement of the denture opposite the side in action. As the lower jaw is drawn into position, the buccal cusps of the lower molars traveling into the sulcus and toward the lingual cusps of the upper molars on the side in use, we find the buccal cusps of the opposite lower molars traveling toward the buccal cusp of the upper molar on the side not in use, keeping a a constant contact for the purpose of supporting the denture upon that side of the mouth during mastication on the opposite side. The same principle holds good when mastication is performed on either side of the mouth. Fig. 2 shows the position of molars and

Fig. 1.

Fig. 4.

Fig. 2.

Fig. 5.

Fig. 3.

Fig. 12.

bicuspids during mastication on the right side, Fig. 12 during mastication on left side, with a balancing contact of the molar teeth on the opposide side of the mouth.

The next observation made by Dr. Bonwill has reference to the relative position and size of the teeth in the arch. This is illustrated in Fig. 9. An equilateral triangle is formed with a base corresponding to a line drawn from the center of one condyle of the lower jaw to the center of the opposite condyle, and represented by the points "A—A" in the figure. From the points "A—A" a line is taken to the cutting edge of the central incisor teeth at the median line which represents the apex of the triangle, and it is usually about four inches in length. We now take a pair of dividers and obtain the combined width of the superior central, lateral and cuspid teeth on one side. A line is now drawn from the point "F," or the cutting edge of the superior central incisors at the median line, to a point midway between the condyles, or midway between the points "A—A," Fig. 9. This line from "F" to "T" in the diagram represents the median line of the mouth throughout its entire extent. The point of the dividers is now placed at the cutting edge of the superior centrals at point "F," while the other foot of the divider is placed at point "I" on the median line or just the distance of the width of the superior central, lateral and cuspid teeth on one side. The point of the divider resting at "I" is held firmly in position, while the point of the divider at "F" is made free and a complete circle described. The anterior segment of this circle which intersects the median line at "F" gives the exact size of the arch as it falls directly upon the cutting edges of the incisor and cuspid teeth. It marks the extreme limit and prominence of the cuspid tooth. If a line be drawn at right angle to the point where this circle intersects the median line at " Y," (or twice the distance of the combined width of the superior central, lateral and cuspid), from the apex of the triangle, "it will fall through the center of the second molar tooth." After arranging the six anterior teeth according to the measurements given, a straight line is taken from the condyle of one side of the mouth to the distal surface of the cuspid tooth on the opposite side of the mouth. Another line is drawn from the condyle on the opposite side to the cuspid tooth on the opposite side at its distal surface. These lines intersect the median line at "B." Dr. Bonwill claims that a line drawn at right angles to the median line at the point "B" will fall through the center of the first molar tooth. I would say with reference to this line that its position can be slightly altered according as the width of the incisor teeth is greater or less, and also the width of the bicuspid teeth, but the difference is so slight that it makes very little practical difference in the arrangement of an artificial set of teeth. By reference to Fig. 16, which is taken from life-size engraving in Black's Anatomy and enlarged, keeping the proportions as nearly accurate as possible, you will find that the horizontal line falls through the middle of the distal buccal lobe of the first molar tooth, instead of through the center of that tooth.

The arrangement as shown in Black's Anatomy has been more universal in the measurements that I have made than that shown in Dr. Bonwill's diagram.

We now take our dividers and place one point of the divider at "A," and the opposite point of the divider at "B;" we describe a curve toward the buccal surface of the mouth from "B," and we find that this gives us a space between the curve and distal surface of the cuspid tooth, which fixes the width of the first bicuspid tooth. We keep one point of the divider at "A" and we retract the other point to "I;" we describe a second outward curve, and the space between these curved lines gives the exact width of the second bicuspid tooth. The next line is taken from a point at the distal surface of the cuspid tooth to the condyle of the jaw, which Dr. Bonwill claims will pass through the buccal cusps of the bicuspid and molar teeth. In every instance that I have made the measurement, where I have found the arrangement of the teeth approximating the natural or normal, I have found this line to deviate slightly from the diagram shown by Dr. Bonwill. If we refer again to Fig. 16 we find that the two bicuspids and first molars will fall with their buccal cusps upon this line, while the second molar is turned inward toward the

Fig. 16.

median line of the mouth, and is missed entirely by the line from "A" to "C," Fig. 16.

This seems to me to be a more advisable arrangement of artificial teeth, for if the diagram of Dr. Bonwill is followed exactly, the distal buccal cusps of the second molars are thrown too far from the ridge, and there is great danger of excessive leverage at this point, which would not be the case if the second molar was turned toward the median line, as shown in Fig. 16. With the exception of this tooth and the position of the line which defines the center of the first molar I have found no exception to Dr. Bonwill's measurement. There are some other points in connection with this subject which I desire to make, and which I think are of great importance in the arrangement of artificial teeth. I regret exceedingly that Dr. Bonwill did not have more to say in his article on the articulation of the teeth about this point, viz: the angles of force during the acts of incising the food and mastication. When teeth are firmly imbedded in the alveolar process and supported by each other a slight change in the angle of position of these teeth in the process is of very little importance practically, but the angles of the teeth on an artificial denture contribute largely to the successful or unsuccessful use of that denture. The remarks made by Dr. Bonwill have direct application to the support of artificial dentures when the cutting edges of the teeth are in contact or in positive mastication, but there is nothing said about supporting the denture while the food is between the teeth and before the cutting edges strike. It is absolutely necessary to consider the lines of force, or the mechanical forces, during the incisive function and during mastication, in order to support the denture before the cutting edges of the teeth come into actual contact. By reference to diagram "A" I have tried to outline the position of the incisors, bicuspids and molar teeth on opposite sides of the mouth, indicating the lines of pressure during the incisive function and during mastication, by darts. In a large number of cases where there is excessive absorption of the ridges it is a rule with a great many operators to lean the cutting edge of the incisor teeth toward the lip, while the cervix of the tooth is inclined toward the alveolar ridge; the same is true of the lower incisor teeth, and is indicated by Fig. 5. If the mouth is thrown open and there is an attempt to incise with the teeth in this position, the pressure from the lower tooth is against the inner or lingual cutting edge of the upper incisor tooth, and the angle of pressure is, according to the dart, from " B " to " A," which would cause a tilting of the plate at

FIG.6.

FIG.7.

FIG.8.

the heel; because, there being food between the incisor teeth, there is no contact between the molars. An arrangement of this kind, as shown by Fig. 5, would have a tendency, as can be seen by the angle of these teeth, to displace, by tilting, both dentures at the heel. Referring now to Fig. 4 we find that by inclining the cervix of the superior incisor outward and the cutting edge of the inferior incisor inward, that the line of pressure is toward the center of the palate above and against the labial cutting edge of the incisor, thereby supporting the denture during the process of biting through the food. While this arrangement may not be wholly in accordance with a natural denture, and is slightly exaggerated in the figure, the appearance is much better in an artificial set than the same arrangement might be in the natural teeth. The slight change of these angles does not materially effect the appearance of an artificial denture. If we refer now to Fig. 3, which is an arrangement of the bicuspids and molars that usually accompanies Fig. 5, by the leaning in of the cervix toward the alveolar ridge, it can readily be seen that when there is food between the teeth and pressure is exerted, bringing the lower teeth against the upper, that the pressure would be represented by a line from "A" to "B," and that this pressure toward the buccal surface would tend to displace the denture upon the opposite side of the mouth. If we refer now to Fig. 1 we see that the cervix is inclined away from the ridge, while the cutting edges are inclined toward the center of the mouth. If the food be now grasped between the teeth arranged according to this diagram the line of force would be as indicated in Fig. 2; the pressure being upon the buccal surface toward the center of the palate, while in the lower it would be toward the median line of the floor of the mouth, as indicated by the darts.

When the jaw is protruded laterally for the purpose of triturating the food it is gradually retracted, drawn upward and toward the position of occlusion.

Now, until the cutting edges of the teeth strike, the pressure would be toward and against the lingual cusp of the upper molar in the sulcate groove. It will readily be seen that pressure against this point would support, or tend to support, the denture on the opposite side of the mouth. If the angles of the teeth are arranged properly there will be no displacement of the denture while biting through the food until the cutting edges of the teeth strike; there would be no displacement then unless upon the opposite side of the mouth there was no balancing contact. The real act of mastication occurs after the cutting edges of bicuspids and molars strike, and if, at this time, we have contact upon the opposite side of the mouth we find that the denture would be constantly supported. By reference again to Fig. 2 we see the lines of force indicated until the cutting edges of the teeth strike, as the lower jaw is retracted and the buccal cusps of the lower teeth striking the buccal cusps of the upper, the molars and bicuspids begin to travel, the food being pressed in the sulcate groove between the cusps, upward and toward the position of occlusion, the buccal cusps following into the sulcus of the upper tooth, while the lingual cusps of the lower molar travel over the lingual cusps of the upper molar. During all of this time we find the buccal cusps of the lower molar on the opposite side traveling into the sulcus of the upper molar and toward its buccal cusp, keeping a balancing contact on the side opposite the one in action. These points can be, probably, more clearly demonstrated by reference to diagram "E," Fig. 6; here is shown a case of lower central incisors and cuspids with their abraded cutting edges beveled away from the lingual surface toward the labial side of the tooth. We find that the second molar tooth tilts forward owing to a loss of the first molar and bicuspid teeth. This case, which is taken from nature, and of which I have the model here to illustrate, is not infrequently found in practice. In the case in question the gentleman has had several dentures made and was unable to use either of them, for the reason as shown by the arrangement of the incisor and molar artificial teeth. At every attempt to close the mouth when there was food between the teeth the pressure was against the lingual surface of the superior incisor and toward the lip, while the molar tooth antagonizing with the natural lower tooth was inclined at an angle, as illustrated in Fig. 6. It can be readily seen that pressure against the artificial denture in this case is to force it off the ridge; first, to tilt it by pressure against the incisor; secondly, to force the denture from the ridge by pressure against the artificial molar. The second denture was constructed after the plan of Fig. 7. Instead of inclining the artificial molar it was placed nearly upon a perpendicular plane, while the cutting edge was sloped away toward the distal surface in order to antagonize the tooth. The arrangement of the incisors in this plate was as indicated in Fig. 7. The upper incisor swinging clear of the lower tooth and allowing the lower incisor teeth to strike against the base plate of the denture. In this case the same conditions would hold as in Fig. 6. The line of force is from the distal portion of the plate toward the front of the mouth, and the denture was constantly thrown off the ridge. The manner after which we constructed our appliance, and one which has been worn quite successfully, is shown in Fig. 8. The long projecting inner extension of the lingual surface of the incisor was ground away, as indicated in Fig. 8. It is impossible and quite unnatural to adjust an artificial denture with an overbite and with cusps, in such a case as illustrated in Fig. 8. The cutting edges of the lower natural incisors were slightly grooved, as shown by the figure; while the molar tooth, the first superior molar artificial, was arranged practically as shown in Fig. 7, but in addition to this we arranged the second molar upon the denture, bringing a bearing against the distal surface of the lower natural molar. The provision of this arrangement is quite obvious; during biting upon the front teeth the line of pressure was against the labial cutting edge and toward the center of the palate, as shown by the darts, and while the denture was thus supported anteriorly at the same time of occlusion, in the region of the molars we find the pressure exerted in both directions, and by the tilting of the second molar against the distal surface of the lower natural molar we find the pressure exerted in a manner that would hold the denture firmly against the ridge and prevent its displacement. It is a strange feature that a subject of so great importance has been so long neglected in the literature of mechanical dentistry. The article of Dr. Bonwill's on this subject: "the Articulation of Artificial Teeth," is the first and only literature of any scientific merit in this direction, and after an experience of over six years carefully following his methods of articulating artificial teeth we are prepared to state that our results have been universally satisfactory. If we expect to recover from the present state into which mechanical dentistry has fallen it must be by the study and adoption of such theories as those advanced by Dr. Bonwill. While there may be some criticism as to the conclusions upon the observations he has made, the profession is certainly deeply indebted for such a perfect guide to the articulation of artificial teeth. The subject is of too great importance to be passed without serious consideration, and the moment the intelligent part of the dental profession begin to adopt and apply, carefully, such methods, or require their mechanics to adopt them, they will at once raise the status of the prosthetic art, and a mechanical dentist will be recognized as a scientific man. With the observations before us, it remains to adopt some apparatus by which we can study and obtain the various movements of the lower jaw out of the mouth.— As we have seen by the preceding that the overbite of the incisor teeth, the position of the teeth in the arch, the vertical curvature in the bicuspid and molar region, the length of the cusps, all have a definite relation to each other and cannot be followed abstractly. One cannot hope to carry out the provisions of this articulation without having the means of accurate measurement. We have the vertical curvature in the molar region, of what service is this curvature unless proportioned properly to the overbite? If we place the incisor teeth, as many operators do, with a very slight overlap, "or only enough to prevent hissing," as the books tell us, we at once sacrifice to a large extent the incisive function of the incisor teeth; not only this, but the appearance of the incisor teeth is quite unnatural. If the incisor teeth are arranged regardless of the position of the molars and the natural amount of overlap given, then lateral movements of the jaw will be interfered with, and the patient will be restricted simply to the up and down movements of the jaw. We must adopt a means whereby these various conditions can be measured or relatively proportioned out of the mouth, and this cannot be accomplished without being able to obtain the movements of the lower jaw. The only instrument for this purpose is found in the Anatomical Articulator.

N. B.—Nos. 16 and 17 are reproduced from Black's Dental Anatomy, only using enough of original figure to show relation and position of the teeth.

Nos. 9, 11, 14 and 15 are reproduced from original drawings by Dr. Bonwill as they serve completely the purposes of the paper.

In a subsequent issue the "Anatomical Articulator," and how to use it, will be discussed, when further allusion will be made to the engraving of this issue, and it should therefore be preserved.

## Figures and Tables

**Fig. 9. f1:**
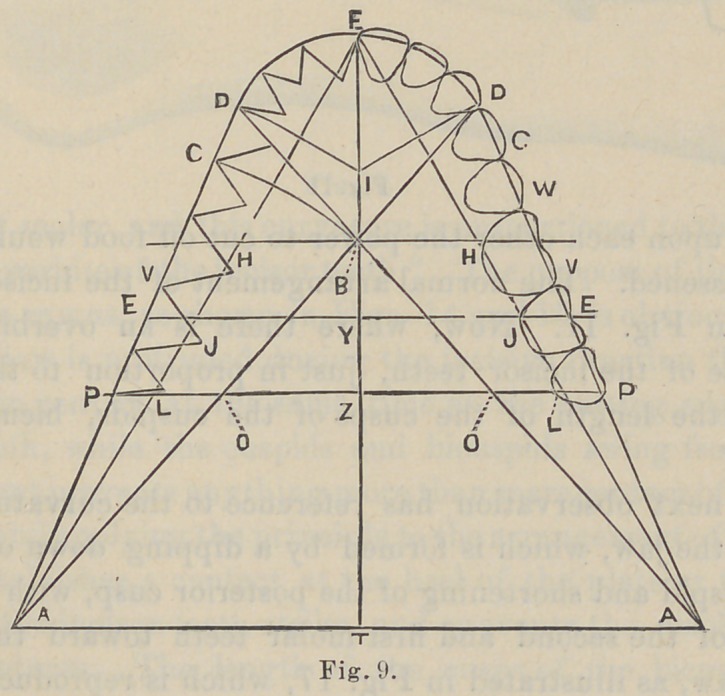


**Fig. 11. f2:**
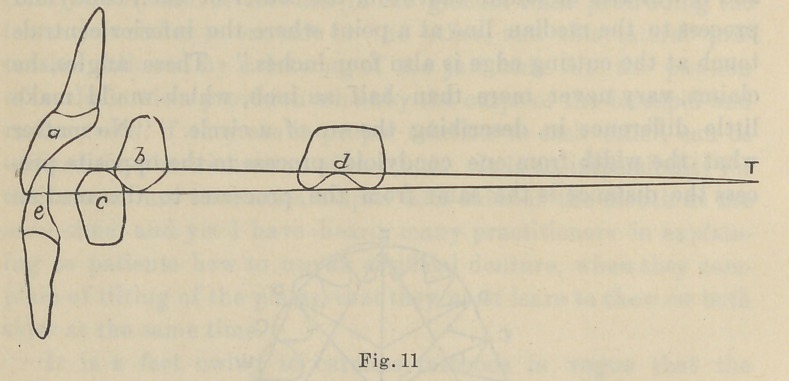


**Fig. 17. f3:**
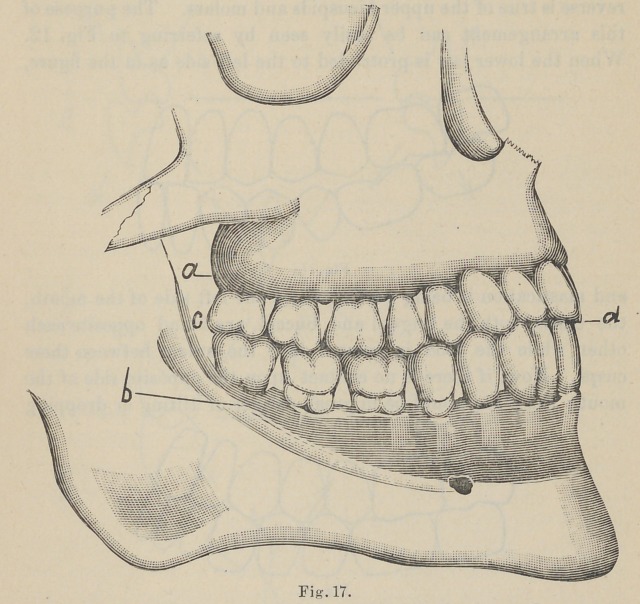


**Fig. 14. f4:**
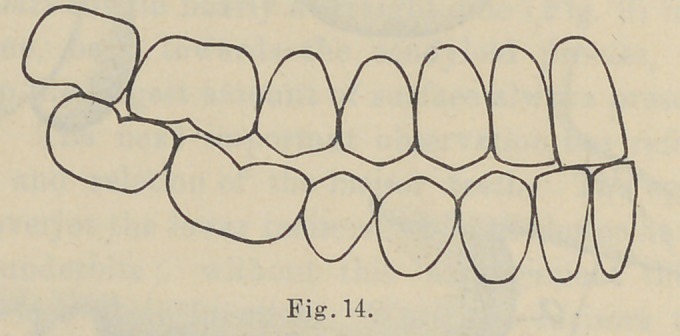


**Fig. 15. f5:**
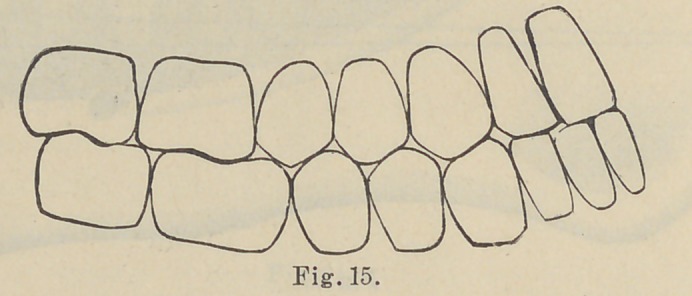


**Fig. 1. f6:**
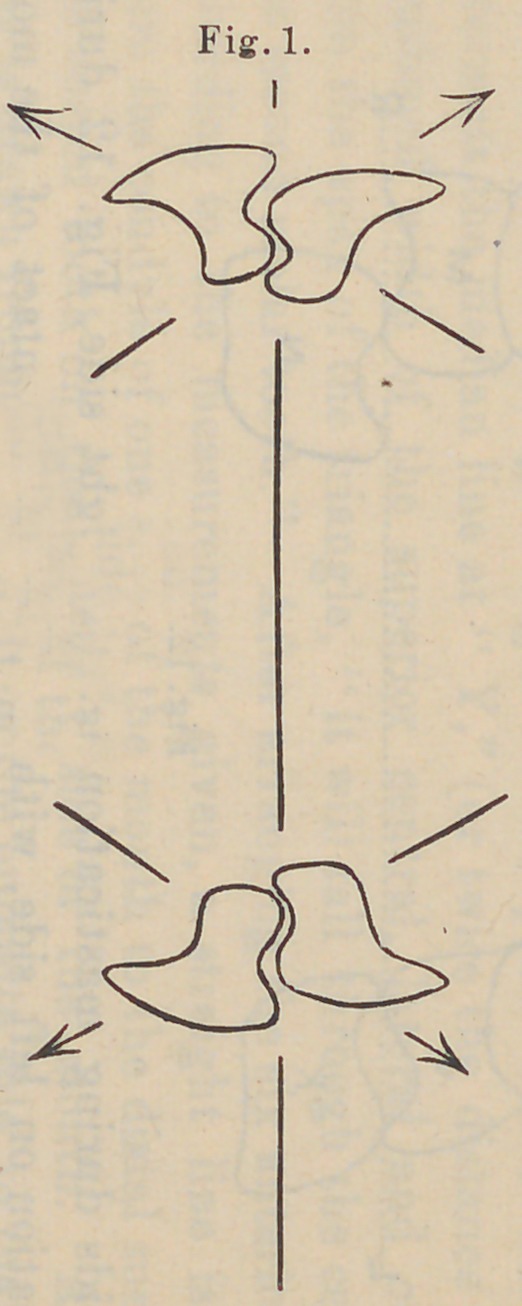


**Fig. 4. f7:**
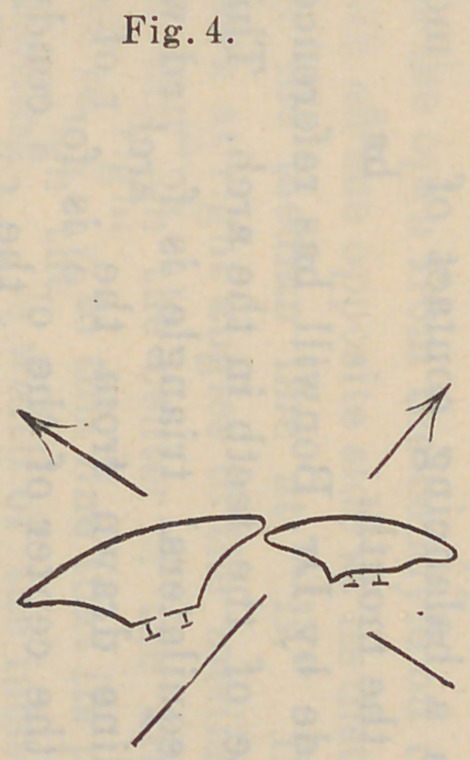


**Fig. 2. f8:**
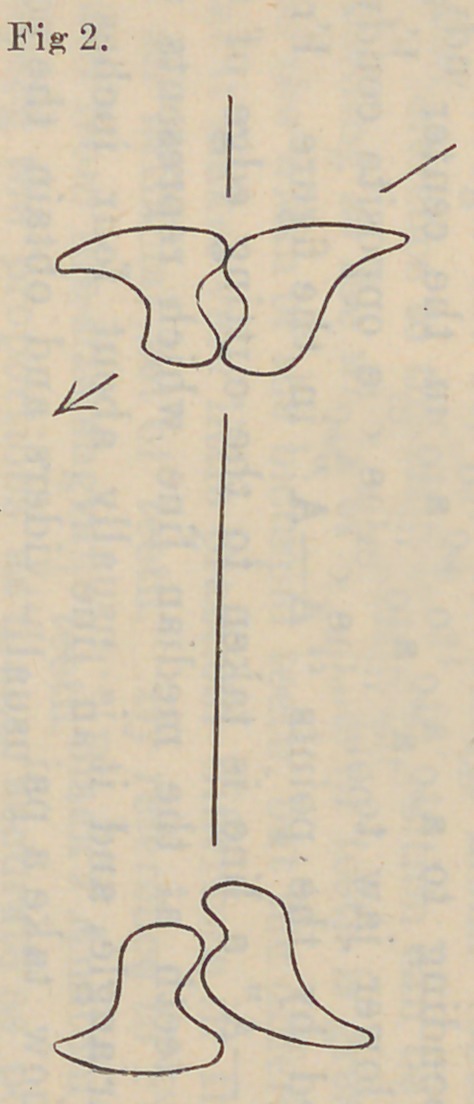


**Fig. 5. f9:**
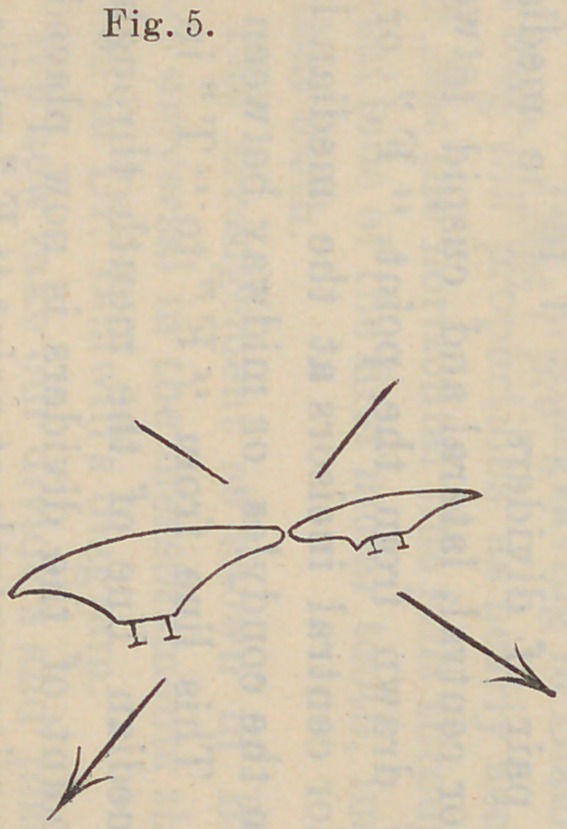


**Fig. 3. f10:**
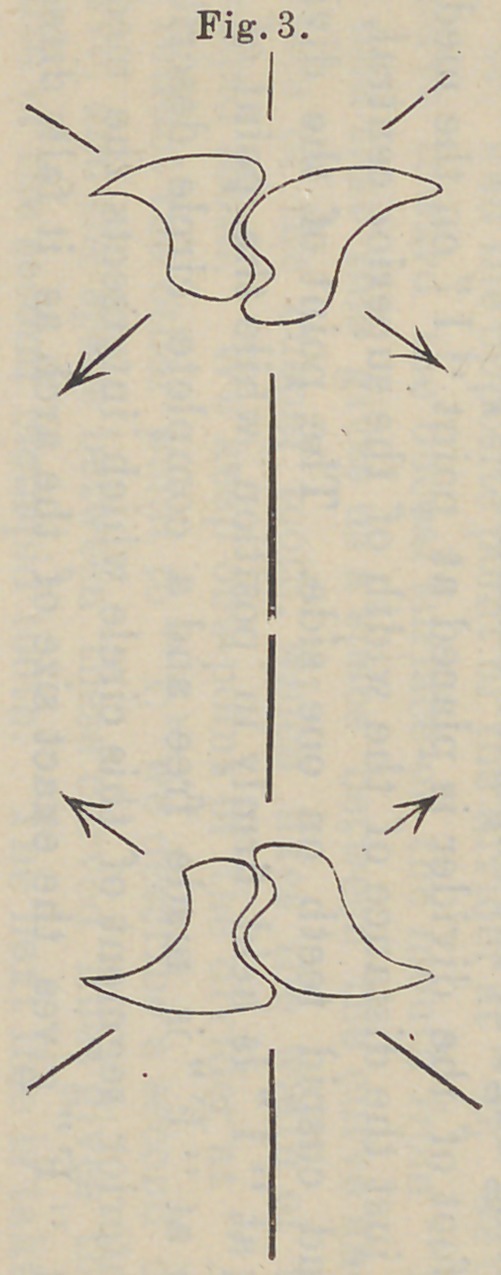


**Fig. 12. f11:**
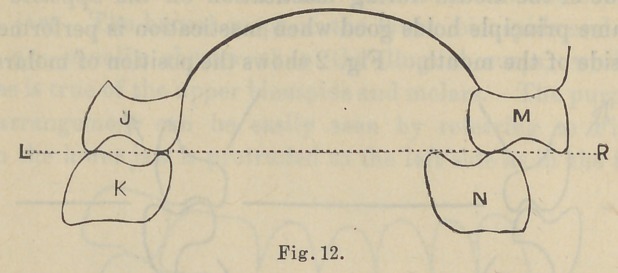


**Fig. 16. f12:**
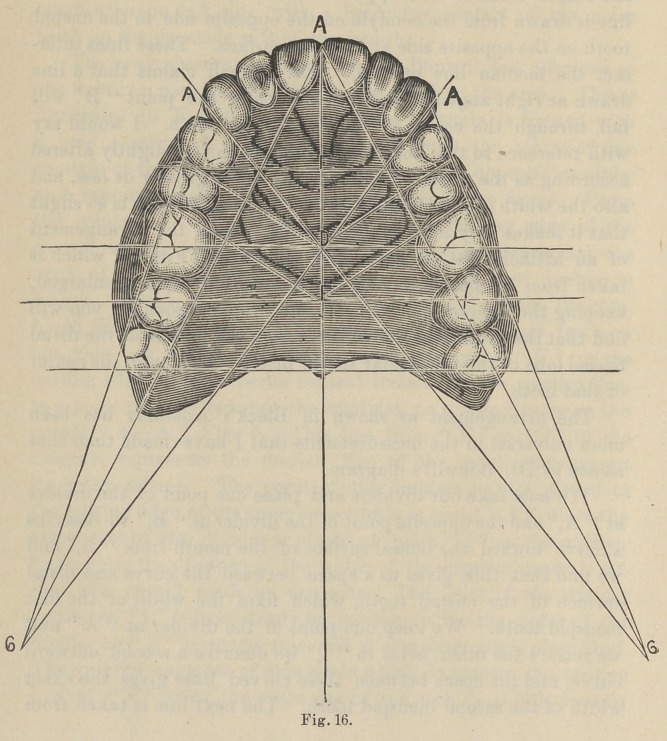


**Fig. 6. f13:**
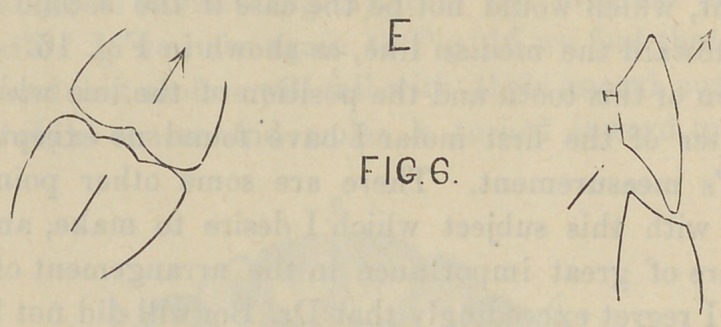


**Fig. 7. f14:**
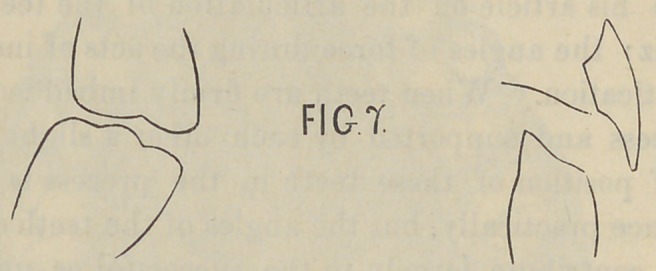


**Fig. 8. f15:**